# Intervention for critical aortic stenosis in Hutchinson-Gilford progeria syndrome

**DOI:** 10.3389/fcvm.2024.1356010

**Published:** 2024-04-25

**Authors:** Leslie B. Gordon, Sammy Basso, Justine Maestranzi, Elena Aikawa, Cassandra L. Clift, Antonio Giovanni Cammardella, Tommaso Hinna Danesi, Pedro J. del Nido, Elazer R. Edelman, Abeer Hamdy, Sheila M. Hegde, Monica E. Kleinman, Nicola Maschietto, Mandeep R. Mehra, Srinivasan Mukundan, Francesco Musumeci, Marco Russo, Frank J. Rybicki, Pinak Bipin Shah, William A. Suarez, Kelsey Tuminelli, Katherine Zaleski, Ashwin Prakash, Marie Gerhard-Herman

**Affiliations:** ^1^ Division of Genetics, Department of Pediatrics, Hasbro Children's Hospital and Warren Alpert Medical School of Brown University, Providence, RI, United States; ^2^ Department of Anesthesiology, Critical Care and Pain Medicine, Boston Children's Hospital and Harvard Medical School, Boston, MA, United States; ^3^ The Progeria Research Foundation, Peabody, MA, United States; ^4^ Associazione Italiana Progeria Sammy Basso, Tezze sul Brenta, Vicenza; ^5^ CNR - National Research Council of Italy, Institute of Molecular Genetics Luigi Luca Cavalli-Sforza, Unit 9 of Bologna, Bologna, Italy; ^6^ IRCCS Istituto Ortopedico Rizzoli, Bologna, Italy; ^7^ Cardiovascular Division, Brigham and Women's Hospital, Boston, MA, United States; ^8^ Department of Cardiac Surgery and Heart Transplantation, San Camillo Forlanini Hospital, Roma, Italy; ^9^ Department of Surgery, Division of Cardiac Surgery, Brigham and Women's Hospital, Boston, MA, United States; ^10^ Department of Cardiac Surgery, Boston Children's Hospital and Harvard Medical School, Boston, MA, United States; ^11^ Institute for Medical Engineering and Science, Massachusetts Institute of Technology, Cambridge, MA, United States; ^12^ Department of Pediatrics, Division of Pediatric Cardiology, Medical College of Georgia, Augusta University, Augusta, GA, United States; ^13^ Cardiovascular Division, Department of Medicine, Brigham and Women's Hospital and Harvard Medical School, Boston, MA, United States; ^14^ Department of Cardiology, Boston Children's Hospital and Harvard Medical School, Boston, MA, United States; ^15^ Department of Radiology, Brigham and Women's Hospital, Boston, MA, United States; ^16^ Department of Medicine, Boston Children's Hospital, Boston, MA, United States; ^17^ Department of Radiology, University of Arizona - Phoenix, Phoenix, AZ, United States; ^18^ Division of Pediatric Cardiology, Department of Pediatrics, University of Toledo Medical Center, Toledo, OH, United States

**Keywords:** aging, aortic stenosis, apico-aortic conduit, atherosclerosis, progeria, transcatheter aortic valve replacement

## Abstract

Hutchinson-Gilford Progeria Syndrome (HGPS) is an ultra-rare genetic premature aging disease that is historically fatal in teenage years, secondary to severe accelerated atherosclerosis. The only approved treatment is the farnesyltransferase inhibitor lonafarnib, which improves vascular structure and function, extending average untreated lifespan of 14.5 years by 4.3 years (30%). With this longer lifespan, calcific aortic stenosis (AS) was identified as an emerging critical risk factor for cardiac death in older patients. Intervention to relieve critical AS has the potential for immediate improvement in healthspan and lifespan. However, HGPS patient-device size mismatch, pervasive peripheral arterial disease, skin and bone abnormalities, and lifelong failure to thrive present unique challenges to intervention. An international group of experts in HGPS, pediatric and adult cardiology, cardiac surgery, and pediatric critical care convened to identify strategies for successful treatment. Candidate procedures were evaluated by in-depth examination of 4 cases that typify HGPS clinical pathology. Modified transcatheter aortic valve replacement (TAVR) and left ventricular Apico-Aortic Conduit (AAC) placement were deemed high risk but viable options. Two cases received TAVR and 2 received AAC post-summit. Three were successful and 1 patient died perioperatively due to cardiovascular disease severity, highlighting the importance of intervention timing and comparative risk stratification. These breakthrough interventions for treating critical aortic stenosis in HGPS patients could rewrite the current clinical perspective on disease course by greatly improving late-stage quality of life and increasing lifespan. Expanding worldwide medical and surgical competency for this ultra-rare disease through expert information-sharing could have high impact on treatment success.

## 1 Introduction

Hutchinson-Gilford Progeria Syndrome (HGPS) is an ultra-rare, fatal, premature aging disease caused by an autosomal dominant single base mutation in the lamin A/C (*LMNA*) gene encoding the nuclear membrane protein lamin A (1, 2). The resulting abnormal protein, called progerin (3), causes phenotype onset shortly after birth, with lifelong failure to thrive resulting in late teenage heights and weights of around 1.2 m and 18 kg, respectively (4); total alopecia; sclerodermatous skin changes (5); abnormal dentition (6); generalized lipodystrophy; skeletal dysplasia with micro-retrognathia (7, 8); joint contractures (9) and strokes (10). Without intervention, progressive premature atherosclerosis (11–14) results in death at age 14.5 years on average (15).

**Figure 1:**
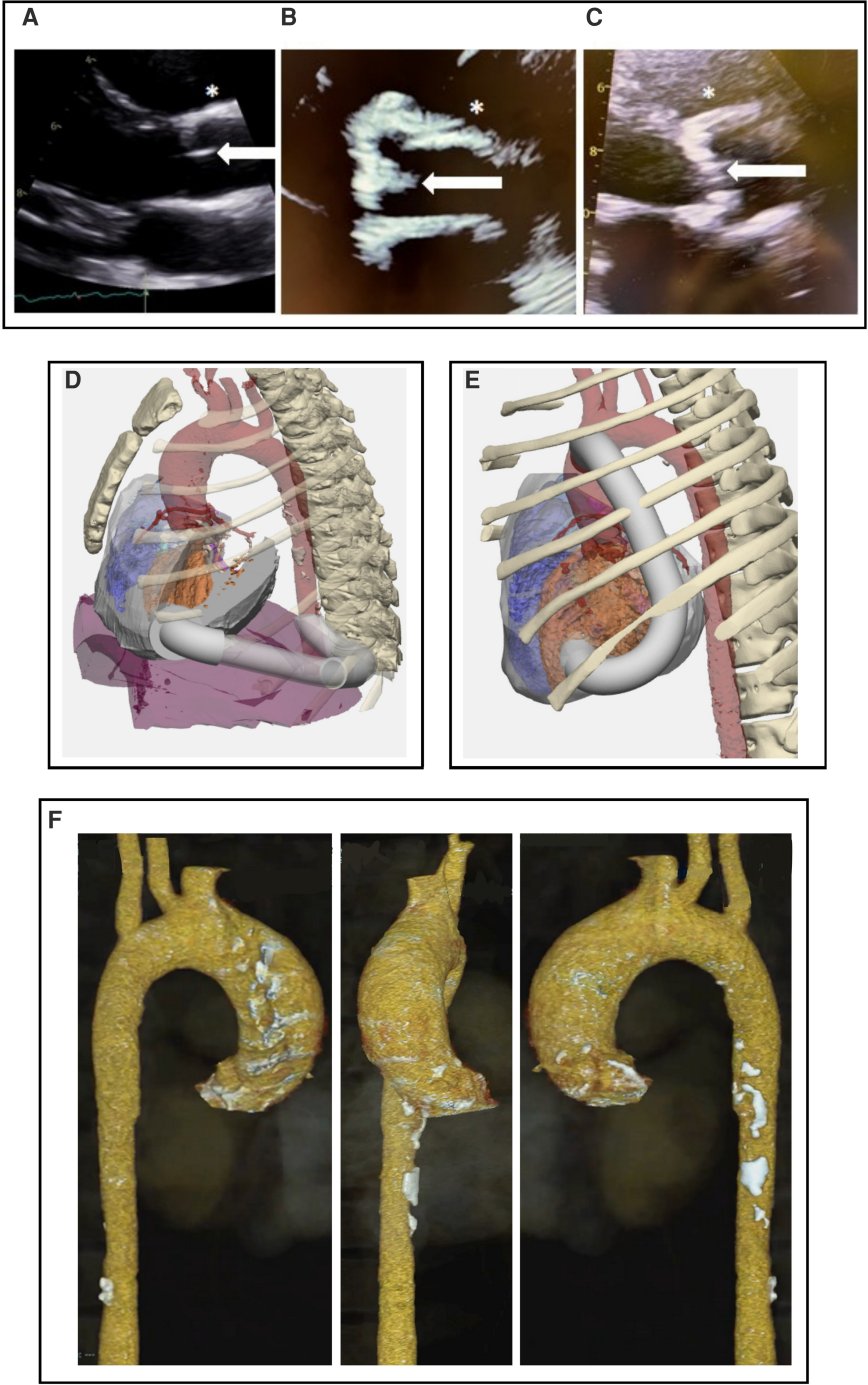
(**A–C**) Parasternal long axis echocardiographic view at the level of the aortic valve. (**A**) 19 y.o healthy control. (**B**) 22 y.o with HGPS and advanced aortic stenosis. (**C**) 72 y.o. with calcific aortic stenosis, demonstating calcific appearance in both aging and HGPS which is unlike the congential aortic stenosis seen in children. Note bright, diffusely thickened aortic valve (white arrows) and aortic wall (*) in (**B**) and (**C**) compared to (**A**). (**D,E**) 3-dimensional CT with AAC modeling performed prior to surgical intervention for cases #3 and #4. Design with conduit (white) origin at cardiac apex and insertion into (**D**) descending aorta and (**E**) ascending aorta. Orange is left ventricle; red is aorta. (**F**) CT 3D reconstruction of case #3 demonstrating calcification throughout the aortic arch and ascending aorta.

Atherosclerosis in HGPS has the hallmark arterial stiffening and fibrocalcific plaques seen in the elderly (11, 12, 14), but develops without obesity, hypercholesterolemia, diabetes or smoking (16, 17). Figures 1A–C demonstrates similar pathological appearance between the calcific aortic stenosis seen with both HGPS and aging. Non-HGPS individuals accumulate vascular progerin with increasing age, but at much lower levels than HGPS; this could serve as a common driver of atherosclerosis between HGPS and generalized aging (3, 12).

The only FDA approved treatment for HGPS is the farnesyltransferase inhibitor lonafarnib, which extends lifespan an average of 4.3 years (18, 19) (30%), alongside improving vascular distensibility. As more patients live into their late teens and early twenties, the meeting organizers (L.B.G., A.P., M.G.H.), who examine patients longitudinally from over 40 countries as part of HGPS clinical trials (18, 20), have observed that calcific aortic stenosis (AS) is now emerging as a principal determinant of mortality (21, 22). Its development is rapid, becoming critical within a few years of detecting aortic valve calcification, as opposed to a decade or more in the elderly (11, 14).

With several identified HGPS patients facing mortality related to critical calcific AS and no clear treatment options, the Progeria Aortic Stenosis Interventional Summit was convened. This interdisciplinary conference included participants capable of envisioning and performing innovative percutaneous and surgical intervention. The overarching goal was to develop novel management approaches with the potential to significantly improve quality of life and extend lifespan in this unique patient population. Participants included subject matter experts in HGPS pediatric and adult cardiology, cardiac intervention, neuroradiology, and critical care (see author list). Topics included the HGPS phenotype, its natural history of critical AS, creative strategies for approaching interventional and/or surgical repair, and anticipated disease-specific intra- and perioperative challenges.

## 2 Intervention strategies designed around 4 patient cases

A series of 4 representative cases of classic HGPS with critical AS were used to facilitate discussion. Table 1 includes anthropometric and imaging data salient for evaluating procedures that might be applicable to different patient scenarios, using modifications to the standard non-HGPS adult-based approaches. Major considerations were aortic valve annulus size, cusp dimensions and coronary height. In all cases, ilio-femoral access was ruled out in favor of transapical access due to small arteries and transverse arch curvature, vascular stiffness and plaque obstruction causing increased risk of stroke due to plaque mobilization.

**Table 1:** Key characteristics of calcific aortic stenosis in cases of HGPS^a^.

Case #; sex	1; M	2; M	3; M	4; F
Genetic mutation in LMNA	p.1824 C > T	p.1824 C > T	p.1824 C > T	p.1824 C > T
Body weight (kg)	20	21	15	15
Height (m)	1.36	1.38	1.07	1.14
Duration of lonafarnib therapy (y)	11	6	13	15
Age AS first detected (mild) (y)	18	16	11	16.5
Age at intervention (y)	23	23	15	19
Peak aortic gradient measured prior to pre-intervention value below (mm Hg; # mo. prior to presurgical)	80;9	120;9	55;4	22;9
Last Echocardiographic Findings Prior To Intervention				
Peak aortic gradient (mm Hg)	91	106	84	130
Mean aortic gradient (mm Hg)	51	60	44	79
LV mass/BSA (g/m^2^)	111.9	163.2	166.9	122.2
LV mass *z*-score	4.6	8.1	6.6	4.8
LV ejection fraction	84%	69.5%	76.4%	72.9%
LV lateral E’ velocity (cm/s)	5.9	4.9	6.4	8.5
LV lateral E’ velocity *z*-score	−4.4	−5	−4.2	−3.6
LV septal E’ velocity (cm/s)	5.3	3.2	4.2	6.7
LV septal E’ velocity *z*-score	−3.7	−4.5	−4.4	−3.2
LVH Severity	Moderate	Severe	Severe	Severe
LV Systolic Function	Normal	Low normal	Normal	Normal
LV Diastolic Dysfunction	Severe	Severe	Severe	Severe
Aortic Stenosis Severity	Critical	Critical	Critical	Critical
Aortic valve Regurgitation Severity	Mild	Mild	Mild	Moderate
Mitral valve Stenosis Severity	Mild	Moderate	Mild	None
Mitral valve Regurgitation Severity	None	None	Mild	Mild
Key Echocardiographic Findings After Intervention				
Months after intervention	17	N/A	19	14
Peak aortic valve gradient (mm Hg)	34	N/A	35	36
Mean aortic valve gradient (mm Hg)	18	N/A	19	24
LV ejection fraction	65%	N/A	64.2%	60.5%
Mitral valve regurgitation severity	Medium	N/A	Mild	Mild
Contrast Enhanced Computed Tomography Findings Just Prior to Intervention				
Mean aortic valve annulus diameter (mm)	18.4	17.2	16.0	14.0
Minimum aortic valve annulus diameter (mm)	16.1	14.8	15.1	11.9
Maximum aortic valve annulus diameter (mm)	20.6	19.5	16.7	17.5
Aortic annulus area (mm^2^)	280	228	201	167
Aortic valve perimeter derived diameter (mm)	18.5	17.3	16.2	15.1
Annulus perimeter (mm)	58.6	54.1	50.9	47.4
Aortic root diameter (mm)	22.3	22.1	19.4	16.8
Sinotubular junction diameter (mm)	19	17	18.0	16.1
Sinotubular junction height (mm)	11.4	12.4	9.6	13.1
Ascending aorta diameter (mm)	23	19	24	21
Right coronary ostia height (mm)	8	11	6.6	8.0
Left coronary ostia height (mm)	5	9.6	5.6	7.9
Aortic annular calcification	Severe	Severe	Severe	Severe
Minimum femoral artery diameter (mm)	3.5	3.4	3.6	2.5

N/A, not applicable.

^a^ Key parameters for grading: Mitral valve stenosis defined as mean pressure gradient (mm Hg): Mild ≤ 5, Moderate = 5–12, Severe ≥ 12; LVH is defined as LV mass/BSA (g/m^2^): Mild = 103–116 (M) and 89–100 (F), Moderate = 117–130 (M) and 101–112 (F), Severe ≥ 131 (M) and >113(F); Normal LV systolic function = ejection fraction of 55%–70%; Diastolic dysfunction defined as septal or lateral E′ velocity *z* score < −2.

Cases #1–3 described themselves as asymptomatic, despite extremely high peak gradients with exponential increases over several months, and gradual decline to minimal physical activity (case #3 was wheelchair-bound due to hip instability); case #4 complained of occasional postprandial chest discomfort/angina pectoris, similar to other published cases (23, 24).

### 2.1 Cases undergoing transcatheter aortic valve replacement (TAVR)

Cases #1 and #2 had were of similar age, sex and size; both had minimum allowable aortic valve sizes amenable to the smallest available TAVR. However, patient risk was higher for case #2, where aortic valve failure was evident from declining peak gradient and declining left ventricle (LV) function, and left ventricle hypertrophy (LVH) was more extensive and asymmetric.

Major risks of TAVR were identified as acute annular rupture due to bulky LV outflow tract calcification, coronary obstruction due to low lying coronary arteries, and severe paravalvular leak. Although neither case satisfied standard criteria for TAVR due to close proximity of coronary ostia to the aortic annulus (25), modifications were employed to deal with HGPS size and disease-specific cardiovascular pathology. The balloon-expandable Sapien3Ultra 20 mm bovine valve was employed using a transapical approach through a left mini-thoracotomy. To prevent coronary ostia occlusion, a lower implantation depth was chosen, with prophylactic wiring and undeployed stent placement in the coronary arteries. Immediately post-placement, echocardiography demonstrated optimal TAVR function with no prosthetic paravalvular leak and good flow between the valve and left coronary artery.

The patients experienced drastically different outcomes, likely primarily due to the different stages of AS and concurrent heart disease at the time of the procedure. In Case #1, the intervention was successfully performed, without cardiac sequelae (22). Immediate post-operative aortic gradient was 9 mm Hg (from pre-TAVR of 90). At 3-year follow-up the patient remained asymptomatic with no cardiovascular events. Echocardiographic assessment showed preserved LV ejection fraction (60%) with a reverse remodeling of ventricular wall thickness (11 mm) which decreased from 16 mm pre-TAVR. The aortic gradient increased to 19 mm Hg, possibly caused in part by initial degeneration of the prosthetic valve or continued progression of HGPS pathology. No paravalvular leaks were detected. There is continued longitudinal monitoring.

Follow-up Case #1 patient interviews revealed improved quality of life. In the weeks immediately following TAVR, he noted improved skin color, and a general feeling of wellness not felt in several years. At six weeks post-TAVR, energy level doubled, and physical activities were much easier. This has continued for three years. The patient recognized diminished pre-TAVR status only after TAVR.

In Case #2, a small left thoracotomy was performed and after pericardial opening the patient experienced sudden ventricular fibrillation in the setting of severely reduced diastolic function and reduced LV filling. The presence of moderate mitral valve stenosis further reduced diastolic filling in the setting of extremely hypertrophic LV. The patient was managed with inotropic support and fluid administration with partial recovery. TAVR was successfully deployed with no paravalvular leak and good coronary perfusion. Post-operative LV did not regain function. With inotropic support, the blood pressure increased temporarily but could not be sustained. Temporary intra-aortic balloon pump was not feasible due to small peripheral vessel size. Sternotomy for central extracorporeal membrane oxygenation was performed. The patient developed a severe bleeding coagulopathy and cardiac tissue became rigid, noncompliant, with minimal contractility. The patient died 4 days post-TAVR.

### 2.2 Cases ineligible for TAVR: potential for apico-aortic conduit

Cases #3 and #4 were younger, 25% lighter, with smaller aortic annuli than cases #1 and #2 (Table 1). They were ineligible for the smallest available TAVR (20 mm) due to their small aortic valve annulus sizes and coronary heights. Among surgical options, Ross operation would require extensive decalcification of the aortic annulus and a substantial period of aortic cross clamping. Given the extensive peripheral and cerebrovascular atherosclerosis in HGPS, this would carry high risk of end organ injury. A simpler surgical alternative using an Apico-Aortic conduit (AAC) was explored (26). In this procedure, a prosthetic conduit system relieves the obstruction to LV outflow by adding a second outflow with a bioprosthetic valve from the apex of the LV to the aorta. Most blood flow then bypasses the native valve and exits the heart through the implanted, valved conduit. This decreases the pressure gradient across the native aortic valve, though some blood continues to flow from the heart through the native aortic valve. This invasive procedure usually requires a brief period of cardiopulmonary bypass, but avoids the risk of coronary artery obstruction, and presents a significant advantage over open-heart surgery by avoiding myocardial ischemia from aortic cross-clamp.

In HGPS, the chest cavity is extremely small and pyriform, with a small heart and vasculature. Appropriately sized prosthesis and bioprosthetic valves for AAC are not commercially available, as these special conduits are no longer being manufactured since TAVR was introduced. However, on-site construction of a reinforced expanded Dacron or polytetrafluoroethylene (ePTFE) apical connector possibly stabilized with a flange placed on the external conduit wall, along with a bioprosthetic valved tube graft system is feasible.

Using CT of Case #3, a 3D reconstruction of the chest cavity with the simulated conduit position in the chest was done for purposes of surgical procedure planning (Figures 1D,E). The ribcage was included in the 3D model, to evaluate potential relationship between the apical component of the conduit and the chest wall for conduit placement. Several possible conformations were entertained, such as going around the diaphragm and entering straight posterior. The CT scan showed calcified atherosclerotic plaque in the descending aorta (Figure 1F), so an area in the ascending aorta was evaluated for the distal conduit connection from the apical insertion. Most non-HGPS cases employ a posterior low thoracotomy for insertion of the aortic component of the graft, with conduit tubing run up to the apex (27). However, the benefits of a median sternotomy over thoracotomy to optimize access to the aorta during AAC was felt to outweigh the risk of slow bone healing with rib fracture (7, 8), as better access would minimize overall procedure time and risk of perfusion-related injury. An entryway would be created through the apex, where the conduit would be inserted and grafted. A major consideration is the ability of patients to tolerate an estimated 30–45 min of cardiopulmonary bypass and how the hypertrophic and fibrotic HGPS myocardium would respond to the ischemic injury from cross clamp. Theoretically, everything could be done with a beating heart, however, due to the need to partially clamp the ascending aorta, a brief period of cardiopulmonary bypass but with heart beating mitigates risk.

Because the conduit system is separated from the native aortic valve, the newly inserted valve can be larger than the native valve aperture. Simultaneously, the flexible conduit can be smaller in diameter than the valve itself, to accommodate its insertion into the small sized ventricle. This optimizes flow capacity and maintains a low gradient across the new valve. The smallest available prosthetic valve is a 12mm porcine valve with a 12 mm connection into the LV. This valve comes pre-mounted on a synthetic tube graft of the same diameter and has historically performed well, without experiencing elevated gradients or prosthesis mismatch even in growing children. It was estimated that this size would reduce the peak aortic gradient in the native vessel from 120 to 30 mm Hg, with at least 50%–70% of blood flowing through the conduit. Importantly, the remaining 30%–50% of flow across the native aortic valve and to the coronary arteries, along with low-dose aspirin therapy, mitigates the risk of aortic thrombosis.

Importantly, the component of the graft that is inserted into the apex of the LV must be rigid enough to prevent compression from the cardiac muscle contraction surrounding it. Options to address this design requirement are to insert the actual valve with its support stent at the apical end of the conduit, place a fully stented valve such as the Melody valve within the conduit, or recreate the reinforced synthetic tube to mimic the design of the original AAC device that is no longer available. Considerations for choosing among these options were the location of the LV apex with respect to the chest wall, particularly since these are very hypertrophied hearts in thin chests, the need to place the proximal end of the conduit far enough inside the LV to prevent tissue covering the orifice and to aim the proximal end away from the ventricular septum, which could also restrict inflow into the conduit. Based on these considerations, an elbow design and a proximal end to the conduit that had sufficient reinforcement to prevent compression was felt to be ideal. Thus, a two-part conduit was needed, containing both an elbow section with a long enough tubular segment to enable positioning well inside the LV, and a second section which would be made of flexible graft containing the prosthetic valve in the center. The synthetic graft would need to be stiff enough to resist kinking as it coursed from the LV to the aorta, over the lung towards the aorta yet flexible enough to permit direct anastomosis to the ascending aorta. The Hancock conduit (Medtronic) was chosen for the second component of the composite conduit because the Dacron graft is stiff enough to resist kinking, particularly when filled with blood at aortic pressures, while at the same time allowing for creation of an anastomosis to the aorta.

Risks of various methods for cardiopulmonary bypass were considered. Whereas normally for left heart surgical procedures a left atrial to aortic bypass is sufficient to permit manipulation of the LV, in these severely obstructed hearts, significant manipulation of the LV required by creation of an apical defect to accept the proximal conduit and suturing of the conduit and flange greatly increases the risk of inducing ventricular fibrillation. Additionally, to create the distal conduit anastomosis in the aorta, a partial occluding clamp that occludes a large enough section of the aorta to connect the 12 mm tube graft would be required and this step could restrict coronary blood flow sufficiently to induce ischemia and arrhythmias. Therefore, it was felt that full cardiopulmonary bypass to support the right and left heart would be the safest and most expeditious approach.

Pre-surgery modeling using individual patient data will be critical for appropriate surgical readiness (Figures 1D,E). The 3D-model of the chest cavity would ideally include both the model shown, and a full phase model, including ventricular contraction and complete heartbeat. A simulation that includes all of this information would demonstrate complete flow dynamics using various conduit options. For example, case #3 might require a more lateral conduit positioning because the echo of this patient revealed a cavity obliteration that could in turn result in a conduit obliteration.

Following this summit, cases #3 and #4 underwent AAC surgery successfully, though due to aortic calcifications in the descending aorta, the case #4 conduit was inserted into the ascending aorta (Figure 1E) (28). Median sternotomy healed well and there were no postoperative complications.

### 2.3 Balloon valvuloplasty as an adjunctive treatment

The main risks to balloon angioplasty are ischemic stroke and new aortic regurgitation (29). However, because a small increase in circumference has a relatively large effect on flow capacity across the valve, balloon angioplasty only decreasing the gradient a small amount may be considered as a temporary mitigating strategy while planning for additional intervention, if TAVR or AAC cannot be implemented in the near term.

### 2.4 Non-procedural HGPS-specific considerations for intervention success

Airway management: Due to severe retrognathia, a small and narrow nasal pathway, unusual glottic angle, and decreased neck mobility (30, 31), supraglottic airways and fiberoptic intubation techniques are often needed if laryngoscopy is not possible, and airway equipment sizes should not be based on the age, but on height.

Protecting blood flow: For cardiovascular protection with advanced atherosclerosis, and to avoid stroke in the face of intracerebral vessel deterioration and collateral vessel formation (10), hypovolemia, hypoperfusion, and medications or anesthetic agents that can increase myocardial oxygen consumption or that can result in hypotension should be avoided (30). Pre-operative discontinuation of low dose aspirin therapy may increase the risk of thrombosis (32). Finally, extreme lipodystrophy (33) increases the risk of hypothermia, which should be carefully monitored and addressed.

## 3 A patient perspective

The meeting participants included one patient-scientist with HGPS who not only underwent TAVR (case #1) (22) but also experienced association with the patient in case #2 who did not survive the TAVR. This young man described his perceived lack of symptoms pre-TAVR and his dramatic increase in quality of daily life post-TAVR. As a young adult facing a first-ever high-risk intervention for HGPS, he was aware that his life expectancy without intervention was less than one year, but that the risks associated with intervention (i.e., stroke, death) could cut that year dramatically short. This patient, like so many others with HGPS, has spent a lifetime watching his friends with HGPS pass away, and hence has developed a strong awareness of his life's fragility and value. He described his experiences with others who view life extension in HGPS as a futile attempt to prolong a life fraught with serious illness. His view strongly opposed this rhetoric. When presented with the surgical option, he felt that the procedure was his “last opportunity to be involved in the world”. Understanding the risks, he chose to undergo the TAVR. He explained that his extensive involvement with researchers and physicians spanning 15 years of clinical treatment trials taught him that prolonging his life and the lives of all children with HGPS, even for a day, is a gift. His desire was to send a message that “everyone should understand that we are alive; that HGPS does not prevent us from living a happy life, and we want to continue to experience this life”. Further, he stated, “I strongly believe that the discussions within this meeting are a real turning point for the HGPS patient community. Now, not only can we see the research community developing drugs that will create a future cure for new generations of children born with progeria, but we can also have new hope today that an international group of doctors will be ready to intervene on those of us with critical AS”.

## 4 Conclusions

HGPS is an ultrarare genetic disease with a dramatically shortened lifespan subsequent to accelerated premature atherosclerosis. However, more patients are living longer with lonafarnib therapy (18, 19). Consequently, this summit group identified the emergence of an older segment of this population in their mid-teens to early 20s experiencing critical calcific AS, the pivotal end-stage yet potentially treatable event. Both healthspan and lifespan could be increased, possibly by years, with successful intervention focused on alleviating calcific AS. Although a systematic study in the younger (median age 12 years) lonafarnib-treated HGPS population demonstrated aortic calcification preceding stenosis at rates of 36% and 10%, respectively (13), Progeria Aortic Stenosis Interventional Summit findings underscore the need for robust evaluation of AS in older aged patients.

With the unique disease-specific mosaic of challenges in mind, this group framed and implemented two new nonstandard avenues for surgical intervention in HGPS based on 4 representative cases. Although all had similar phenotypes including extremely small body habitus, global lipodystrophy, bone dysplasia, advanced systemic atherosclerosis and neurovascular disease, each differed in key clinical factors that necessitated the development of alternative strategies for intervention: TAVR and AAC, each performed in nontraditional ways to accommodate patient needs.

### 4.1 TAVR discussion

Only the largest patients with HGPS will be eligible for the smallest currently available artificial aortic valve (4). Generation of a smaller aortic valve would greatly expand the number of children eligible for TAVR, which is less invasive than AAC surgery. Smaller TAVRs would allow for earlier intervention when risks are lower, as well as benefiting smaller patients with HGPS at late stages of calcific AS.

Proximity of coronary arteries to the annulus is key to determining feasibility of valve replacement. In HGPS, the coronary ostia are low and close to the aortic valve; thus, they are highly susceptible to occlusion with current valve implantation and expansion. Also, coronary protection during the intervention is essential. In both cases, the aortic valve was implanted lower than is customary, to prevent coronary ostia obstruction; this increases the risk of heart block but makes the intervention possible.

### 4.2 AAC discussion

Patients for whom the currently available aortic valves are too large should be considered for AAC surgery. Though appropriately small AAC systems are not commercially available, they can be custom fabricated *de novo* at major medical centers based on fully modeled 3-dimentional chest CT, similar to the cases presented. A principal consideration is evaluating the need for cardiopulmonary bypass while maintaining adequate blood flow to the heart and brain, given the accompanying global atherosclerosis.

### 4.3 Optimizing risk/benefit ratio with cardiovascular monitoring and timing of intervention

Timing of intervention is a pivotal issue, given the HGPS-specific risk factors and limited surgical experience in these patients. Intervening too early will risk shortening lifespan prematurely. Conversely, patients at late stages with failing hearts have little time remaining to perform intervention; waiting more than a few months may significantly increase the risk of perioperative death, as in case #2.

Echocardiography is recommended annually from birth, increasing in frequency once LVH or AS is detected. When peak aortic gradient reaches 20 mm Hg, more frequent echocardiography and intervention planning should be initiated. Late accelerated progression of AS permits only a small window for optimizing risk/benefit ratio before failure ensues. The cases presented here suggest a peak aortic gradient between 80 and 90 mm Hg as a rough guide to intervention timing, keeping in mind that the gradient will increase exponentially with time at later stages of disease.

### 4.4 Overall importance

HGPS does not affect cognition, giving those affected by this disease the capacity to both understand their prognosis and develop rich lives filled with joy. The several years of life extension due to lonafarnib therapy has produced a growing population of young adults with HGPS whose main barrier to survival is critical AS. Though there are many associated risks to intervention, the opportunities afforded by extending both healthspan and lifespan using the modified TAVR and AAC interventions for critical AS discussed in this summit meeting, when anticipated life expectancy is less than one year, are invaluable.

Given that HGPS is an ultra-rare disease (34), many patients will not have access to the essential expertise for successful AS intervention. This summit was intended to stimulate critical early evaluation and prospective planning for intervention through extensive communication among HGPS experts and interdisciplinary medical teams at sites of intervention both pre-and post-operatively. Only by combining efforts worldwide will the HGPS community have the best chance of meeting this urgent unmet clinical need.

## Data Availability

The original contributions presented in the study are included in the article/Supplementary Material, further inquiries can be directed to the corresponding authors.
